# Prior metabolic surgery attenuates the weight-loss efficacy of liraglutide in patients with mild obesity

**DOI:** 10.3389/fendo.2025.1580159

**Published:** 2025-05-22

**Authors:** Yuqin Ouyang, Xinyue Xiang, Xinyun Hu, Xuehui Chu, Wenjuan Tang, Wenhuan Feng

**Affiliations:** ^1^ Department of Endocrinology, Endocrine and Metabolic Disease Medical Center, Nanjing Drum Tower Hospital Clinical College of Nanjing University of Chinese Medicine, Nanjing, China; ^2^ Branch of National Clinical Research Center for Metabolic Diseases, Nanjing, China; ^3^ Department of General Surgery, Drum Tower Hospital Affiliated to Nanjing Medical University, Nanjing, China; ^4^ Department of Endocrinology, Endocrine and Metabolic Disease Medical Center, Affiliated Drum Tower Hospital, Medical School, Nanjing University, Nanjing, China

**Keywords:** glucagon-like peptide-1 receptor agonist, metabolic surgery, weight loss variability, body mass index, metabolic adaptation

## Abstract

**Background:**

Liraglutide effectively manages mild obesity, but individual weight loss outcomes vary significantly. We aimed to identify clinical predictors influencing differential treatment responses in patients with mild obesity.

**Methods:**

A retrospective analysis was conducted on 64 adults (BMI 28–32.5 kg/m²) undergoing a 12-week liraglutide intervention. Participants were categorized based on therapeutic success: those achieving composite endpoints (≥5% total weight loss [TWL] and BMI normalization to <28 kg/m²) versus suboptimal responders. Comprehensive biometric and biochemical assessments were performed, and multivariate predictive modeling was applied.

**Results:**

Responders (n=37, 75.7% female) showed significantly better metabolic outcomes than non-responders (n=27, 77.8% female), with notable differences in %TWL (11.0 ± 3.6% vs 4.2 ± 2.6%), total weight loss (9.04 ± 3.32 kg vs 3.55 ± 2.20 kg), and BMI reduction (3.3 ± 1.1 vs 1.4 ± 0.9 kg/m²) (all p’s <.01). Responders also demonstrated improved glucolipid metabolism, and reduced metabolic-associated fatty liver disease (p <.05). Regression analysis identified a history metabolic surgery (MS) and a baseline BMI ≥30.5 kg/m² as significant negative predictors of success. Adjusted odds ratios indicated strong inverse associations, with MS history showing an OR of 6.78 (95% CI: 1.95–23.61; p <.01) and elevated BMI (≥30.5 kg/m²) yielding an OR of 4.79 (95% CI: 1.46–15.71; p <.01).

**Conclusion:**

A history of MS significantly affects liraglutide’s responsiveness in patients with mild obesity, emphasizing the need for personalized therapeutic strategies in post-surgical patients. These findings highlight the importance of a comprehensive medical history in guiding obesity pharmacotherapy.

## 1 Introduction

Mild obesity (body mass index [BMI] 28–32.5 kg/m²) is a significant contributor to the obesity epidemic in China, posing risks for metabolic complications such as metabolic dysfunction-associated steatotic liver disease (MASLD), cardiometabolic disorders, and progression to moderate-severe obesity (BMI ≥32.5 kg/m²) if untreated (1). Cohort studies suggest that a 5–10% total weight loss (%TWL) in this group can significantly reduce obesity-related complications, making weight management the first-line therapeutic approach (1, 2).

Glucagon-like peptide-1 receptor agonists (GLP-1RAs), including liraglutide, have revolutionized obesity management through their therapeutic efficacy, achieving an average of 8.0% weight loss in phase III clinical trials (3). However, individual weight loss outcomes vary (3–5), and the factors influencing this variability are not fully understood. Identifying predictors of liraglutide’s weight-loss efficacy could optimize its therapeutic use.

For patients with moderate to severe obesity, metabolic surgery offers significant benefits by promoting substantial weight loss and improving metabolic health. These procedures typically function by restricting food intake, altering nutrient absorption, or both (6, 7).​The most commonly performed procedure is sleeve gastrectomy (SG), which involves removing approximately 80% of the stomach to create a tubular “sleeve,” thereby reducing stomach capacity and decreasing hunger hormone (ghrelin) production. Another prevalent procedure is the Roux-en-Y gastric bypass (RYGB), which creates a small stomach pouch and reroutes the small intestine to limit both food intake and nutrient absorption. biliopancreatic diversion with duodenal switch (BPD-DS) combines a sleeve gastrectomy with a significant bypass of the small intestine, leading to substantial weight loss and metabolic improvements, particularly in type 2 diabetes management. However, BPD-DS carries a higher risk of nutritional deficiencies, making it less commonly performed (6–8). SG is associated with a lower perioperative complication rate compared to RYGB and demonstrates comparable efficacy in weight loss and improvement in metabolic indicators within the initial years post-surgery (8). Consequently, SG has become increasingly popular, representing a growing proportion of metabolic surgeries (8). ​ However, long-term outcomes may favor RYGB concerning sustained weight loss and metabolic benefits (6–8). A newer, simplified procedure, the single-anastomosis duodenoileal bypass with sleeve gastrectomy (SADI-S), which utilizes a single intestinal connection to reduce surgical complexity while maintaining efficacy. Studies indicate that SADI-S achieves comparable or superior weight loss outcomes to RYGB and SG, with fewer long-term complications (9). These surgical options are tailored based on individual patient profiles, considering factors such as BMI, comorbidities, and previous surgical history.

With the growing prevalence of metabolic surgery (MS), a significant number of patients post-MS experienced weight regain, leading to a recurrence of mild to moderate-severe obesity if not managed promptly. Adjunctive weight loss therapies are increasingly needed for this patient group (10). A meta-analysis of 16 studies (N=881) showed that liraglutide treatment (ranging from 3 months to 4 years) resulted in a mean weight reduction of 16.03 kg in patients with ≥ 5 years post-MS (11). In patients exhibiting persistent/recurrent type 2 diabetes mellitus (T2DM) post-MS and a baseline BMI ≥37.0 kg/m², liraglutide achieved a mean weight reduction of 5.26 kg over 26 weeks (12). Comparative analyses demonstrated comparable weight reduction between patients post-MS and non-surgical counterparts undergoing liraglutide therapy. Notably, these findings were observed in cohorts with a baseline BMI ≥35 kg/m², a threshold exceeding current therapeutic guidelines for anti-obesity medications (12, 13). Although patients with mild obesity constitute part of the indicated population for liraglutide therapy, it remains unclear whether prior MS influences the observed heterogeneity in weight loss outcomes.

To fill this knowledge gap, we initiated the current research to analyze the response of patients with mild obesity to liraglutide, including those post-MS. The findings indicate that previous MS reduces GLP-1RA efficacy, underscoring the need for tailored therapeutic strategies in this rapidly expanding patient population.

## 2 Materials and methods

### 2.1 Study design

**Figure 1:**
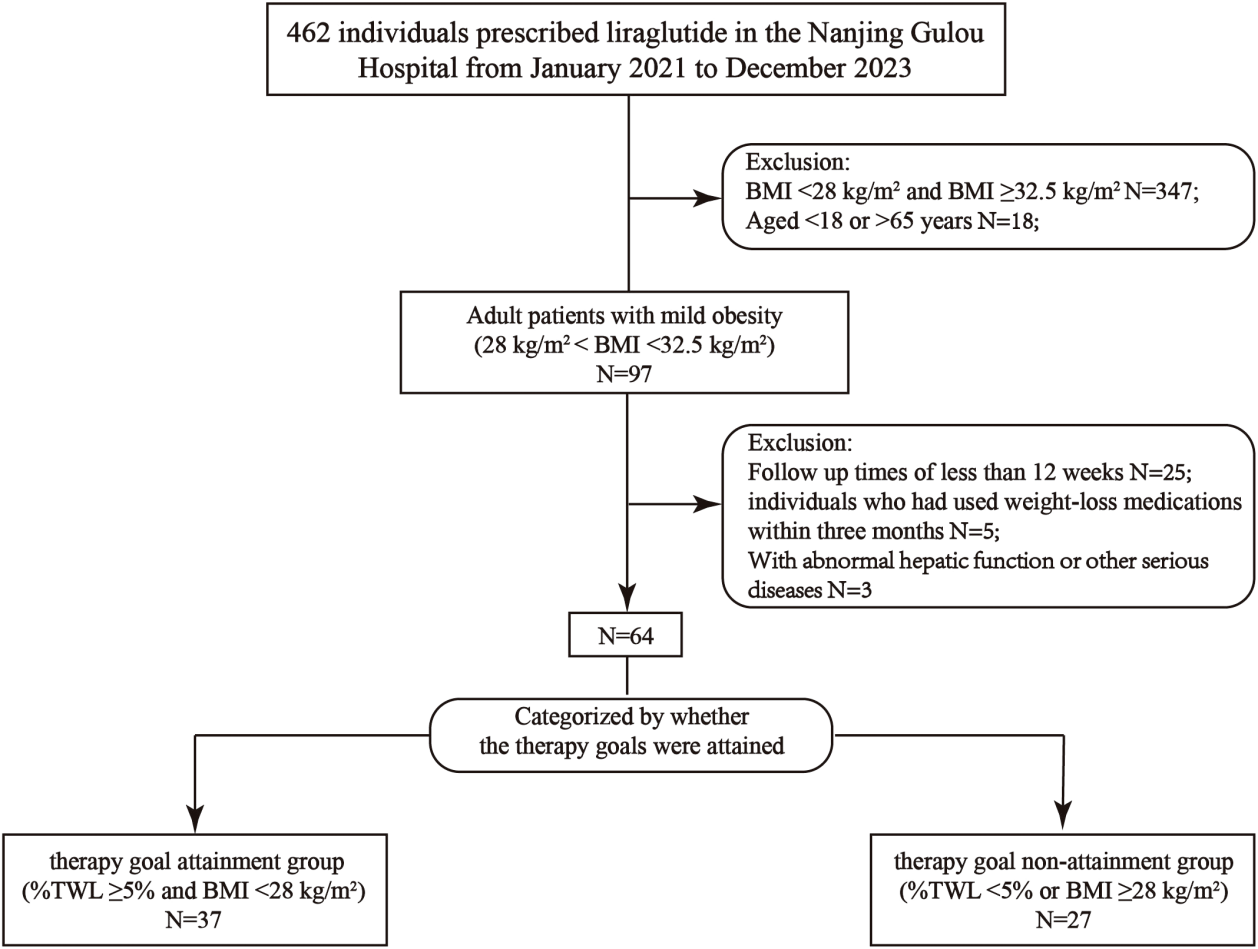
Flow chart of the study.

This retro-cohort study was carried out from February 2021 to December 2023 at Drum Tower Hospital, affiliated with Nanjing University Medical School in Nanjing, China. Adult patients with mild obesity (28 kg/m^2^ ≤ BMI <32.5 kg/m^2^) who completed 12 weeks of liraglutide treatment were enrolled in the present study. Participants were grouped according to the attainment of goals or not after treatment: the goal attainment group (achieved treatment targets of ≥5% total weight loss [%TWL] and BMI < 28 kg/m²) and the goal non-attainment group (did not meet the targets) (**Figure 1**).

Liraglutide dosing 0.6 mg per day in the initial week, increasing to 1.2 mg on week 2, and 1.8 mg per day in the 3rd week until completion of treatment. Weight reduction and alleviation of the obesity-related complications from the two groups were analyzed. Personalized guidance on diet and physical activity was provided to all patients. The dietary intervention targeted an approximate daily caloric reduction of 500 kcal, along with a recommended exercise regimen comprising 150 minutes of moderate-intensity aerobic activity and 60 minutes of resistance training per week. These recommendations were adjusted during follow-up visits based on each patient’s progress and clinical feedback. However, adherence to the prescribed dietary and exercise protocols was not quantitatively assessed.

This study was approved by the ethics committee of Nanjing Drum Tower Hospital (2023-507) and in accordance with the Declaration of Helsinki. All participants had written informed consensus.

### 2.2 Study population

Adult patients with mild obesity who completed 12 weeks of liraglutide treatment were enrolled and received follow-up visits every 4 weeks from the start of treatment with nutritional and physical activity counseling (14).

Patients were excluded if they had used weight-affecting drugs such as GLP-1RAs, sodium-glucose cotransporter protein 2 inhibitors, metformin, orlistat within 3 months before treatment. Additionally, exclusion of individuals with glomerular filtration rate (eGFR) <60 ml/min/1.73 m^2^, alanine aminotransferase (ALT) >100 U/L, aspartate aminotransferase (AST) >100 U/mL, serum procalcitonin levels above the limit of normal, or suffering from cardio-cerebral, psychiatric disorders, malignancies, pancreatitis, severe gastrointestinal disorders, or acute infections.

Type 2 diabetes mellitus (T2DM) was diagnosed following the criteria of the World Health Organization. Remission of T2DM was defined as glycated hemoglobin (HbA1C) level <6.5%, without any anti-diabetes medications for at least 3 months (15, 16).

Hypertension is defined as systolic blood pressure (SBP) ≥140 mmHg, diastolic blood pressure (DBP) ≥90 mmHg, and/or using antihypertensive medications. A blood pressure of 120/80 mmHg was considered remission of hypertension when not on antihypertensive medication (17). MASLD was diagnosed using abdominal ultrasound, and assessed for lipid deposition and hepatic fibrosis by controlled attenuation parameter (CAP) and liver stiffness measurements (LSM) (18). Hyperuricemia is diagnosed by serum uric acid (UA) level ≥420 mmol/L or receiving anti-hyperuricemic drugs. Dyslipidemia is defined by fasting total cholesterol ≥5.2 mmol/L, low-density lipoprotein (LDL) cholesterol (LDL-C) ≥3.4 mmol/L, high-density lipoprotein (HDL) cholesterol (HDL-C) <1.04 mmol/L, triglycerides ≥1.7 mmol/L, in addition to previous lipid-lowering medication therapy. (19). Relief of dyslipidemia and hyperuricemia was defined as normalization of serum lipid and uric acid levels in biochemical values when not on medication (20).

### 2.3 Data collection

Data covering weight, height, blood pressure, comorbidities, and medications from patients at baseline and every 4 weeks were collected. Lab data consisting of liver and kidney functionality, blood fat levels, glycometabolic markers, and nutrients were measured at baseline and week 12. Static model assessment of insulin resistance (HOMA-IR) on the formula: HOMA-IR = (fasting insulin level × FBG level)/22.5 was calculated at baseline and 12 weeks. (21). Measurement of controlled attenuation parameters and liver stiffness utilizing a FibroTouch^®^ (Haskell Medical Technology Co., Ltd., Wuxi, China). Visceral fat area and body composition on the InBody 720 (BioSpace Co., Ltd., Seoul, Korea) were evaluated.

All participants’ %TWL, Δweight, BMI, and ΔBMI at each follow-up visit for assessment of weight loss on liraglutide. Calculations based on the following formulas: %TWL = ([baseline weight - weight at follow-up]/baseline weight) × 100%; Δweight = (weight at follow-up) - (baseline weight); BMI = body weight (kg)/height^2^ (m^2^); and ΔBMI = (BMI at follow-up) - (baseline BMI) (22).

### 2.4 Statistical analyses

Statistics were analyzed by IBM SPSS Statistics version 26.0 for Windows (IBM Corp.). Categorical variables are described by frequency, and analyzed with Pearson’s χ2 test and Fisher’s exact test. Continuous variables are compared by independent samples t-test. All types of variables are presented by percentages and means ± SD. Linear regression models based on baseline were used to analyze the differences. Differences before and after the intervention between groups were evaluated with paired t-tests. Variations in weight, BMI, and %TWL were examined using repeated-measures ANOVA, and tests for homogeneity of variance were performed prior to conducting ANOVA. To identify factors affecting the weight loss efficacy of liraglutide, multiple stepwise logistic regression was performed.

Paired comparisons were made by *post hoc* tests. Statistical analysis of outcomes was conducted with the t-test and Mann-Whitney U-test. Differences in comorbidities were evaluated by the chi-square or Fisher’s exact test, with standardized residuals for multiple comparisons. Additionally, ordinal logistic regression and multivariate logistic models were employed to determine the factors. Diagnostic accuracy was evaluated with receiver operating characteristic curves (ROCs). A p <0.05 was considered statistically significant for all hypothetical tests.

## 3 Results

### 3.1 Baseline characteristics

A total of 64 patients were included in the study. The mean age of the participants was 35.6 ± 8.2 years, and the mean BMI was 30.0 ± 1.7 kg/m^2^. Of these, 37 were in the goal attainment group (28 females, age: 35.5 ± 8.9 years) and 27 were in the goal non-attainment group (21 females, age: 35.6 ± 7.5 years).

At baseline, the goal attainment group had a lower BMI (29.3 ± 1.2 vs 30.7 ± 1.9 kg/m^2^, *P* <.01), lower serum 25-(OH)-D (16.7 ± 4.8 vs. 21.4 ± 8.7 ng/L, *P* <.05), lower prevalence of hypertension (13.5 vs 77.8%, *P* <.01) and lower prevalence of hyperuricemia (48.6 vs 77.8%, *P* <.05). Additionally, a smaller proportion of patients in the goal attainment group had a history of MS (16.2 vs 55.6%, *P* <.01), and they exhibited higher serum FBG levels (5.3 ± 0.9 vs. 4.8 ± 0.6 mmol/L, *P* <.05) compared to the goal non-attainment group. Other baseline characteristics remained similar in the two groups (**Table 1**).

**Table 1:** Clinical characteristics of enrolled patients at baseline and 12 weeks.

Variables	Goal attainment group (n=37)		Goal non-attainment group (n=27)		P	Estimated Treatment Difference Between Two Groups Mean (95%CI)	P
	Baseline	12 weeks	Baseline	12 weeks	Baseline		12 weeks
Patients’ characteristics							
Age (yr)	35.5 ± 8.5		35.6 ± 7.5		0.96		
Male (%)	9 (24.2%)		6 (22.2%)		0.85		
Post-surgery (%)	6 (16.2%)		15 (55.6%)		<0.01		
SG (%)	5 (83.3%)		13 (86.7%)		0.81		
Anthropometric data							
Weight (kg)	81.3 ± 9.3	72.3 ± 8.1*	84.5 ± 8.6	80.9 ± 8.2*	0.17	-5.9 (-7.3 ~ -4.5)	<0.01
ΔWeight (kg)		9.0 ± 2.0		3.6 ± 2.5		3.2 (-0.8 ~ 11.8)	0.08
BMI (kg/m^2^)	29.3 ± 1.2	26.1 ± 1.4*	30.7 ± 1.9	29.4 ± 1.9*	<0.01	-2.2 (-2.7 ~ -1.6)	<0.01
ΔBMI (kg/m^2^)		3.3 ± 0.3		1.1 ± 0.4		0.5 (1.1 ~ 3.1)	<0.01
%TWL		11.0 ± 3.6		4.2 ± 2.6		6.8 (5.2 ~ 8.5)	<0.01
TWL% ≥5 (n, %)		37 (100%)		12 (44.4%)			<0.01
Waist/Height Ratio	0.57 ± 0.03	0.51 ± 0.03	0.58 ± 0.05	0.55 ± 0.03	0.34	-0.04 (-0.02 ~ -0.06)	<0.01
BFM (kg)	29.9 ± 3.8	23.6 ± 4.0*	31.8 ± 5.5	29.2 ± 4.9*	0.11	-4.1 (-5.2 ~ -2.9)	<0.01
SMM (kg)	28.7 ± 5.4	27.0 ± 5.2*	28.7 ± 4.3	28.1 ± 4.5*	0.97	-1.1 (-1.6 ~ -0.6)	<0.01
SLM (kg)	48.3 ± 8.4	46.3 ± 8.1*	49.6 ± 7.0	48.4 ± 7.3*	0.54	-0.4 (-2.5 ~ -1.1)	<0.01
FFM (kg)	51.3 ± 8.7	48.7 ± 8.2*	52.6 ± 7.6	51.7 ± 7.9*	0.54	-1.7 (-2.6 ~ -0.9)	<0.01
VFA (cm^2^)	143.9 ± 28.1	106.4 ± 32.1*	150.9 ± 32.3	131.6 ± 26.7*	0.44	-19.9 (-32.5~ -7.3)	<0.01
SBP (mmHg)	123.3 ± 11.6	116.9 ± 10.5*	126.2 ± 10.5	126.1 ± 9.6	0.43	-7.8 (-14.1 ~ -1.5)	0.02
DBP (mmHg)	79.4 ± 7.2	76.5 ± 8.3	83.9 ± 9.1	81.6 ± 7.9	0.10	-2.7 (-7.6 ~ 2.1)	0.27
CAP (dB/m)	294.8 ± 23.1	259.2 ± 22.0*	290.7 ± 31.7	277.7 ± 23.6	0.62	-20.1 (-33.2 ~ -6.9)	<0.01
LSM (kPa)	7.0 ± 2.4	6.1 ± 1.6	7.0 ± 1.9	6.8 ± 1.6	0.93	-0.7 (-1.7 ~ 0.4)	0.19
Clinical parameters							
HBA1c (%)	5.4 ± 0.9	4.8 ± 0.6*	5.4 ± 0.5	5.2 ± 0.5	0.52	-0.2 (-0.4 ~ 0.0)	0.04
FBG (mmol/L)	5.3 ± 0.9	4.8 ± 0.6*	4.9 ± 0.5	4.8 ± 0.6	0.03	-0.0 (-0.4 ~ 0.2)	0.52
FIN (uIU/mL)	16.4 ± 8.3	11.3 ± 6.1*	15.5 ± 11.1	14.0 ± 11.0	0.95	-3.1 (-6.8 ~ 0.6)	0.09
HOMA-IR (mmol/L, IU/mL)	4.0 ± 2.4	2.4 ± 1.4*	3.0 ± 1.6	2.5 ± 1.3	0.38	-0.4 (-1.1 ~ 0.4)	0.31
TG (mmol/L)	1.8 ± 1.2	1.1 ± 0.6*	1.3 ± 0.7	1.1 ± 0.6	0.09	-0.1 (-0.4 ~ 0.2)	0.40
TC (mmol/L)	4.5 ± 1.2	4.2 ± 0.9	4.4 ± 1.0	4.3 ± 0.9	0.67	-0.2 (-0.6 ~ 0.2)	0.33
HDL-C (mmol/L)	1.2 ± 0.3	1.2 ± 0.3	1.3 ± 0.3	1.3 ± 0.3	0.13	-0.0 (-0.2 ~ 0.1)	0.46
LDL-C (mmol/L)	2.8 ± 0.9	2.5 ± 0.7*	2.6 ± 0.8	2.5 ± 0.8	0.35	-0.1 (-0.4 ~ 0.1)	0.29
ALT (U/L)	32.8 ± 24.2	20.4 ± 10.4*	23.5 ± 13.9	19.7 ± 9.6	0.08	-0.8 (-5.8 ~ 4.2)	0.75
AST (U/L)	24.9 ± 13.2	19.2 ± 6.5*	19.9 ± 6.4	19.9 ± 7.5	0.08	-1.8 (-5.3 ~ 1.7)	0.32
GGT (U/L)	31.6 ± 24.7	22.1 ± 14.7*	26.4 ± 13.5	23.0 ± 13.4	0.33	-1.8 (-8.9 ~ 5.3)	0.61
UA (mmol/L)	375.2 ± 97.6	332.6 ± 93.0*	366.8 ± 84.9	357.2 ± 79.8	0.59	-30.7 (-62.5 ~ 1.2)	0.06
25-(OH)-D (ng/L)	16.7 ± 4.8	19.9 ± 5.4*	21.4 ± 8.7	22.2 ± 10.0	0.02	2.1 (-0.4 ~ 4.6)	0.10
VB_12_ (pg/mL)	575.0 ± 99.5	540.8 ± 88.2	557.6 ± 91.7	532.0 ± 82.8	0.42	-52.7 (-134.5 ~ 29.2)	0.18
FOL (pg/mL)	14.4 ± 4.7	12.4 ± 5.5	15.5 ± 5.1	18.1 ± 4.1	0.96	-4.3 (-10.0 ~ 1.4)	0.12
ferritin	148.2 ± 91.9	121.2 ± 77.6	66.6 ± 85.5	72.9 ± 79.5	0.07	0.6 (-29.6 ~30.8)	0.97
Obesity-related complications							
T2DM (n, %)	3 (8.1%)	0	2 (7.4%)	2 (7.4%)	0.92		0.10
Hypertension (n, %)	5 (13.5%)	2 (5.4%)	21 (77.8%)	5 (18.5%)	<0.01		0.10
Hyperuricemia (n, %)	18 (48.6%)	10 (27.0%)	21 (77.8%)	8 (29.6%)	0.03		0.82
Dyslipidemia (n, %)	16 (43.2%)	15 (40.5%)	12 (44.4%)	8 (29.6%)	0.93		0.38
MASLD (n, %)	37 (100%)	31 (83.8%)	27 (100%)	27 (100%)	1.00		0.03

*ALT*, alanine transaminase; *AST*, aspartate aminotransferase; *BFM*, body fat mass; *BMI*, body weight index; *CAP,* ultrasound attenuation parameter; *DBP*, diastolic blood pressure; *FBG*, fasting blood glucose; *FCP*, fasting C-peptide; *FFM*, fat-free mass; *FINS*, fasting insulin; *GGT*, gamma-glutamyltransferase; *HbA1c*, glycated hemoglobin; *HDL-C*, high-density lipoprotein cholesterol; *HOMA-IR*, Homeostatic Model Assessment for Insulin Resistance; *LDL-C,* low-density lipoprotein cholesterol; *LSM*, liver stiffness measurement; *MASLD,* metabolic dysfunction-associated steatotic liver disease;*RYGB, Roux-en-Y gastric bypass*; *SBP*, systolic blood pressure; *SG, Sleeve Gastrectomy*; *SMM*, skeletal muscle mass; *SLM*, soft lean mass; *TG*, triglyceride; *TC*, total cholesterol; *T2DM*, type 2 diabetes mellitus; *UA*, uric acid; *VB_12_*, vitamin B12; *VFA*, visceral fat area; *P <.05; compared with baseline within the group.

### 3.2%TWL, Weight loss, ΔBMI, and Body Composition

**Figure 2:**
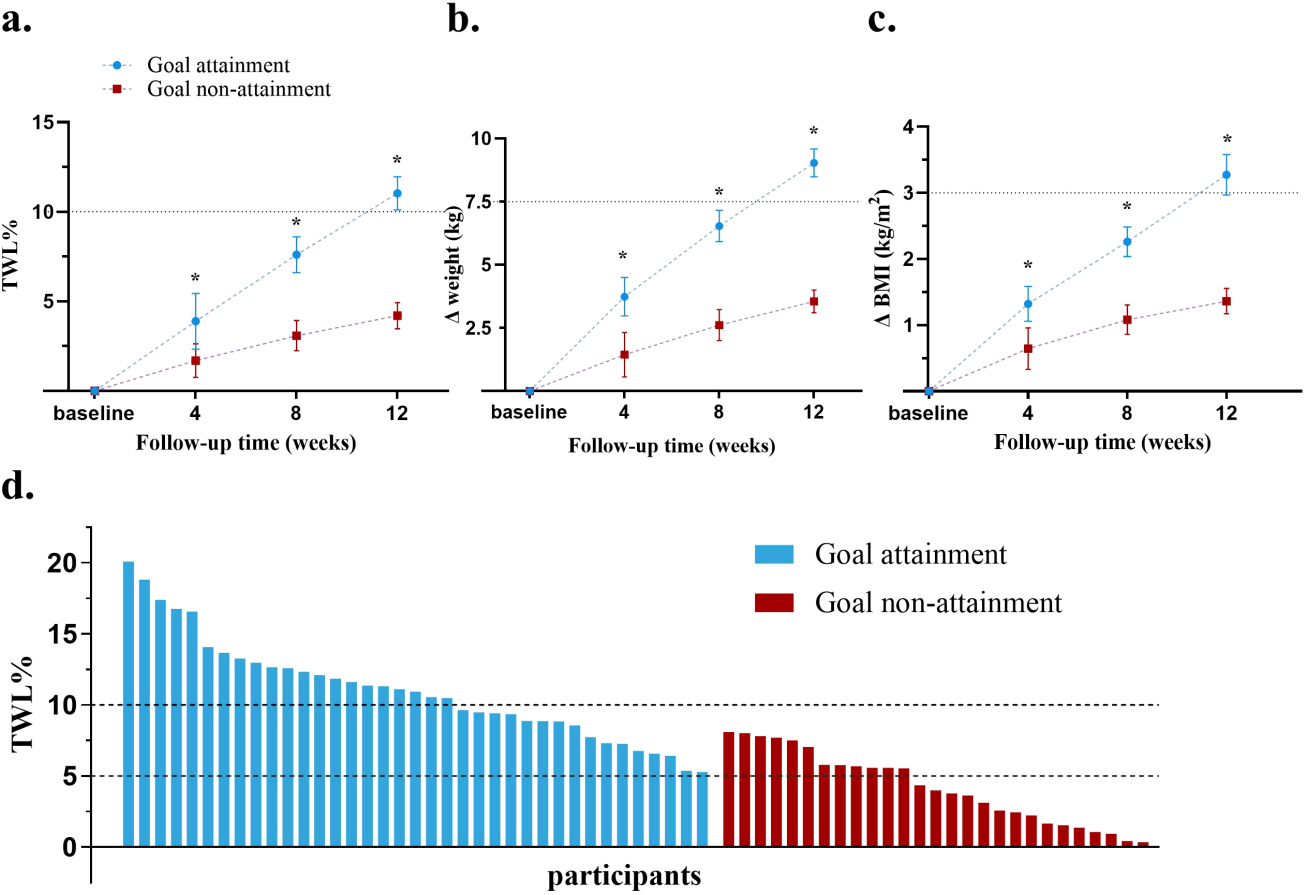
Comparison of weight loss effect in the two groups. **(a)** Changes in mean %TWL at each follow-up; **(b)** Changes in mean weight loss at each follow-up; **(c)** Changes in mean BMI at each follow-up; **(d)** %TWL of each patient after therapy; BMI, body mass index; TWL, total weight loss. *P<0.05.

After 12 weeks treatment, the goal attainment group showed a higher %TWL compared to the goal non-attainment group (11.0 ± 3.6% vs 4.2 ± 2.6%), with an adjusted mean difference of 6.8% (95% confidence interval [CI], (5.2 ~ 8.5)%, *P*<.01). Only 44.4% patients in the goal non-attainment group achieved a %TWL ≥5% (**Table 1** and **Figures 2a**).

Both groups showed reductions in weight and BMI from baseline (*P*<.05 vs baseline). The goal attainment group experienced a significant collective weight loss of 9.0 ± 2.0 kg and a BMI decrease of 3.3 ± 0.3 kg/m^2^. In contrast, the goal non-attainment group showed a weight reduction of 3.6 ± 2.5 kg, resulting in a BMI reduction of 1.1 ± 0.4 kg/m^2^, with no significant change compared with weight and BMI at baseline. The adjusted mean differences in the weight and BMI reductions between the goal attainment and goal non-attainment groups were -5.9kg (95% CI, -7.3 to -4.5, *P*<0.01) and -2.2 kg/m^2^ (95% CI, -2.7 to -1.6, *P*<0.01), respectively (**Table 1**).

Greater %TWL, weight loss, and *Δ*BMI were observed at week 4 in the goal attainment group compared to the goal non-attainment group, which continued to increase over time at weeks 8 and 12 (all *P*<.05) (**Figures 2a**). Waterfall plots illustrated the %TWL for each patient in both groups (**Figure 2**).

**Figure 3:**
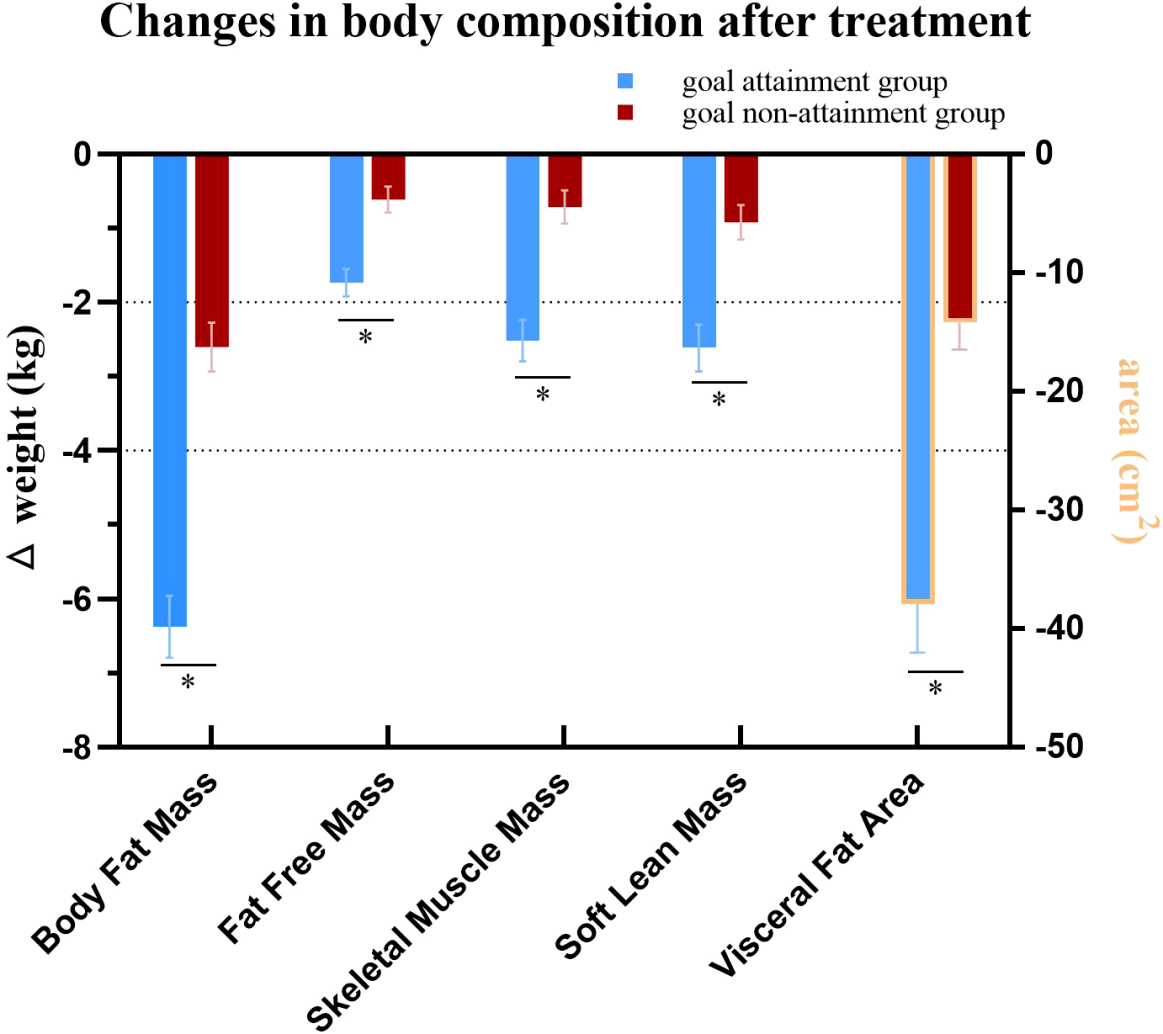
Comparison of changes in body composition after treatment. ^*^ *P*<0.05.

Body composition analysis showed reductions in BFM, SMM, SLM, FFM, and VFA in both groups (all *P*<.05). However, the goal attainment group demonstrated greater reductions in these parameters compared to the goal non-attainment group after 12 weeks of liraglutide treatment (**Table 1**, **Figure 3**).

### 3.3 Remission of obesity-related co-morbidities

Few patients in either group had T2DM. After 3 months of liraglutide treatment, HbA1c, FBG, FIN, and HOMA-IR decreased significantly in the goal achievement group (all *P*<.05), with no changes observed in the goal non-attainment group. Remission of T2DM could not be defined for any patients using liraglutide (**Table 1**). In the goal attainment group, reductions were observed in SBP, serum TG, LDL-C, and UA levels, as well as in the prevalence of MASLD and MASLD-related indicators, including CAP, serum ALT, AST, and GGT levels (all *P*<.05, **Table 1**).

### 3.4 Factors influencing the weight loss efficacy of liraglutide

**Figure 4:**
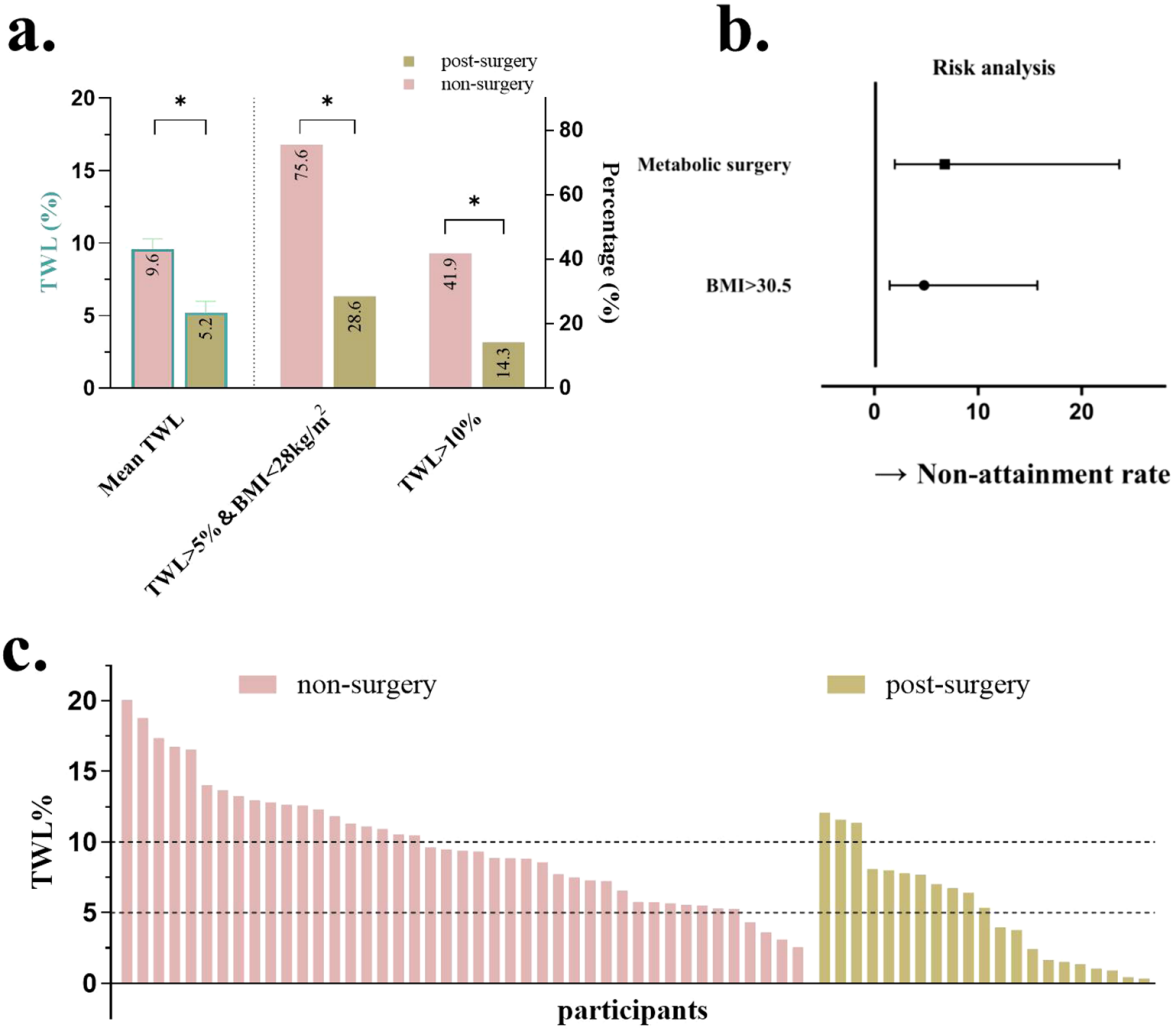
Comparison when grouped by a history of metabolic surgery. **(a)** %TWL after therapy and proportion of patients achieving %TWL>10%, %TWL >5%, and BMI <28; **(b)** Forest plots for the impact of baseline BMI and history of metabolic surgery; **(c)** %TWL of each patient after therapy. TWL, total weight loss; BMI, body mass index. *P<0.05.

Relevant disparities, including baseline BMI, FBG, 25-(OH)-D, MS history prevalence of hypertension, and hyperuricemia were analyzed as variables. The results indicated that BMI and history of MS were significant factors influencing the weight loss efficacy of liraglutide (*P* <.01, **Table 1**). The baseline BMI cutoff of 30.5 kg/m^2^ was calculated from the ROC curve. Forest plots revealed that patients with a history of MS and those with a baseline BMI >30.5 kg/m^2^ were less likely to achieve treatment targets (**Figure 4**).

The area under the ROC curve was 0.715 (95% CI, 0.579–0.851) and 0.697 (95% CI, 0.561–0.832) for baseline BMI and history of MS, respectively (**Supplementary Figure S1**). A baseline BMI cutoff of 30.5 kg/m² was derived from the ROC analysis. As shown in the forest plots, patients with a history of MS and a baseline BMI >30.5 kg/m² were significantly less likely to achieve treatment targets. Adjusted odds ratios (ORs) demonstrated strong inverse associations: a history of MS was associated with an OR of 6.78 (95% CI, 1.95–23.61; *P*<.01), while elevated BMI (≥30.5 kg/m²) was associated with an OR of 4.79 (95% CI, 1.46–15.71; *P*<.01) (**Figure 4**).

Patients were then divided into two groups based on whether or not they had an MS history. Patients without MS had a greater %TWL compared to those who were post-MS (9.6 ± 0.7% vs 5.2 ± 0.8%, *P*<.01, **Figure 4**). More patients without MS history got ≥5% TWL, a BMI <28kg/m^2^, and %TWL ≥10% (75.6% vs 28.6% and 41.9% vs 14.3%, respectively, both *P*<.01, **Figure 4**). Waterfall plots illustrated the %TWL for each patient in groups with and without MS (**Figure 4**).

## 4 Discussion

While achieving %TWL ≥5% serves as a primary therapeutic target in obesity management, our findings suggest that for patients with mild obesity, the dual endpoint of %TWL ≥5% combined with a BMI reduction below 28 kg/m² may better reflect the pharmacological efficacy of anti-obesity medications. In this 12-week liraglutide intervention study, 37 out of 64 participants (57.8%) reached this composite endpoint. Notably, those who achieved the endpoints demonstrated superior therapeutic outcomes compared to non-achievers, including double the %TWL (11.0% vs. 4.2%), nearly three times greater absolute weight reduction (9.0 kg vs. 3.3 kg), and more pronounced improvements in body composition, metabolic parameters, and MASLD.

Multivariate analysis identified a history of MS and a baseline BMI ≥30.5 kg/m² as independent negative predictors of treatment response, potentially due to altered postprandial GLP-1 and PYY secretion patterns in post-MS patients (23). Notably, the diminished efficacy of liraglutide in this population contrasts with previous reports describing enhance GLP-1R effects following bariatric procedures (23). This apparent paradox may reflect adaptive GLP-1R desensitization or downstream signaling alterations resulting from post-surgical metabolic remodeling (24, 25). Furthermore, the type of metabolic surgery performed may modulate GLP-1RA responsiveness by reshaping intestinal anatomy and the spatial distribution of enteroendocrine cells (EECs). As emphasized by Nwako and McCauley (2024), EECs exhibit region-specific and crypt–villus axis-dependent patterns of hormone expression, which are influenced by local signaling gradients and structural remodeling. Surgical procedures such as RYGB, SG, and SADI-S impose distinct anatomical and physiological changes to the gastrointestinal tract, potentially leading to differential enrichment or depletion of GLP-1-, PYY-, or GIP-secreting EECs within the exposed mucosa (26). Although direct assessment of GLP-1R expression was not conducted in this study, prior evidence supports the development of receptor desensitization in response to chronically elevated endogenous GLP-1 levels. Rubio-Herrera et al. observed attenuated pharmacological responses to GLP-1RAs in post-bariatric patients despite high circulating GLP-1 concentrations, likely due to altered receptor density or function (27). Clinical evidence from Shah et al. further demonstrated that GLP-1R blockade using exendin (9-39) markedly reduced endogenous GLP-1 activity within five years post-surgery (24). Supporting this, preclinical models of diet-induced obesity showed blunted GLP-1R-mediated weight loss following experimental MS (25). Taken together, both receptor-level adaptations and region-specific alterations in EEC distribution may collectively contribute to the diminished liraglutide response observed in post-MS individuals.

These findings underscore critical clinical considerations: anatomical and neurohormonal adaptations following MS may require optimized dosing strategies for GLP-1RAs. This concept is consistent with tirzepatide trials (a GLP-1/GIP dual agonist), where activation of the GIP receptor overcame reduced efficacy in treatment-resistant populations (28). Discrepancies with Suliman et al.’s report of comparable liraglutide 3.0 mg/day efficacy between surgical and non-surgical cohorts (13) may stem from differences in baseline BMI (29.8 vs. 37.5 kg/m²), surgical technique (predominantly SG vs. RYGB), and dosing (1.8 mg/day vs. 3.0 mg/day). Our cohort’s lower baseline BMI likely constrained absolute weight loss potential, while subtherapeutic dosing may have further limited responses. Notably, 85.7% of patients in our post-MS patiennts underwent SG, contrasting with Suliman et al.’s cohort where RYGB was the predominant procedure - an important distinction given RYGB’s typically stronger association with weight loss outcomes (13). These differences underscore how post-surgical metabolic adaptations (e.g., sustained appetite suppression and energy expenditure changes) may diminish pharmacological additive benefits (23).

The clinical uncertainty regarding dose escalation to 3.0 mg/day for overcoming metabolic adaptations in mild obesity remains unresolved. This highlights the need for prospective studies to evaluate optimized therapeutic approaches in this population. Clinically, we suggest a practical framework: initiate liraglutide at 0.6 mg/day and titrate weekly to 3.0 mg/day, where approved. Re-evaluate efficacy after 12 weeks at full dose. If <5% TWL is observed, consider next-generation therapies such as semaglutide or tirzepatide (5, 28). Monthly monitoring of weight, body composition (e.g., visceral fat area), and metabolic labs is recommended for response assessment. Prolonged treatment (>24 weeks) may also enhance outcomes via sustained receptor reprogramming (29). Key practical implications include the following: 1) Post-MS patients may require prolonged treatment or higher liraglutide doses to match non-surgical outcomes, consistent with 56-week data showing 3.0 mg’s superiority over 1.8 mg (30); and 2) Dual incretin agonists (e.g., GLP-1/GIP co-agonists) may circumvent reduced GLP-1R sensitivity, as evidenced by recent trials (31).

Our therapeutic success definition (≥5% TWL + BMI <28 kg/m²) revealed baseline BMI ≥30.5 kg/m² as a negative predictor—a finding concordant with global data showing 8.0% mean weight loss with liraglutide 3.0 mg/day in patients without diabetes versus 6.0–4.7% in overweight/obese T2D populations (30, 32). Achieving BMI <28 kg/m² appears particularly challenging with liraglutide 1.8 mg/day in patients with baseline BMI ≥30.5 kg/m². A tiered approach is recommended as follows: begin with dose escalation to 3.0 mg/day for enhanced GLP-1R activation (5); follow with next-generation agents targeting multiple pathways (GLP-1/GIP/glucagon) to bypass metabolic adaptation (27); and consider extended treatment duration (>24 weeks) to maximize neuroendocrine remodeling (33).

The ROC-derived BMI threshold of 30.5 kg/m², slightly exceeding the World Health Organization’s obesity criteria, may mark a pathophysiological inflection point where adiposity begins impairing GLP-1RA pharmacodynamics. Mechanistically, higher BMI is associated with visceral adiposity, chronic inflammation, and potential GLP-1R desensitization (34, 35). Future studies should incorporate body composition metrics (e.g., visceral fat area and waist-hip ratio) to refine stratification (34, 35).

Notably, the goal attainment group showed significant improvements in glucolipid metabolism and MASLD, consistent with the pleiotropic benefits of GLP-1RAs beyond weight loss (36). The metabolic differences between groups may be related to baseline variations in insulin sensitivity, a known modulator of GLP-1RA response (29). However, our regression analysis specifically identified a history of MS, rather than baseline metabolic parameters, as the primary predictor of liraglutide efficacy, emphasizing the importance of considering clinical history in therapeutic decision-making.

This study had some limitations. First, its retrospective design and single-center Chinese cohort limit statistical power and generalizability. Second, local regulatory restrictions capped the liraglutide dose at 1.8 mg/day, only 60% of the approved 3.0 mg/day, which may have reduced efficacy—particularly in post-MS patients with altered incretin signaling. Higher dosing or off-label use may be needed in select populations. Third, adherence-related factors such as social support, care accessibility, and follow-up were not systematically assessed. Fourth, since most patients underwent SG, comparisons across surgical types were not feasible. Given anatomical and physiological differences among SG, RYGB, and SADI-S—which influence enteroendocrine responses and GLP-1RA pharmacodynamics—this is a notable limitation. Lastly, the lack of data on lifestyle and socioeconomic variables, including diet, exercise, and financial constraints, may confound outcome interpretation.In mild obesity post-MS, liraglutide 3.0 mg/day or GLP-1/GIP dual agonists may be required for optimal results. Longer follow-up (≥6 months) is also needed to assess treatment durability.

In conclusion, the majority of patients with mild obesity achieved significant weight loss after 12 weeks of liraglutide 1.8 mg treatment. Our findings support a personalized approach to obesity management, where MS history and baseline BMI ≥ 30.5kg/m^2^ guide GLP-1RA dosing and monitoring. As metabolic procedures and incretin-based therapies increasingly intersect, understanding their bidirectional interactions is essential for optimizing long-term outcomes.

## Data Availability

The raw data supporting the conclusions of this article will be made available by the authors, without undue reservation.

## Supplementary material

Supplementary Figure 1 ROC curve for the impact of baseline BMI and history of metabolic surgery.
