# Supplementary figures and images for: Prior metabolic surgery attenuates the weight-loss efficacy of liraglutide in patients with mild obesity
Source: Front Endocrinol (Lausanne). 2025 May 22;16:1580159. doi: 10.3389/fendo.2025.1580159 (PMC12137074; doi:10.3389/fendo.2025.1580159)

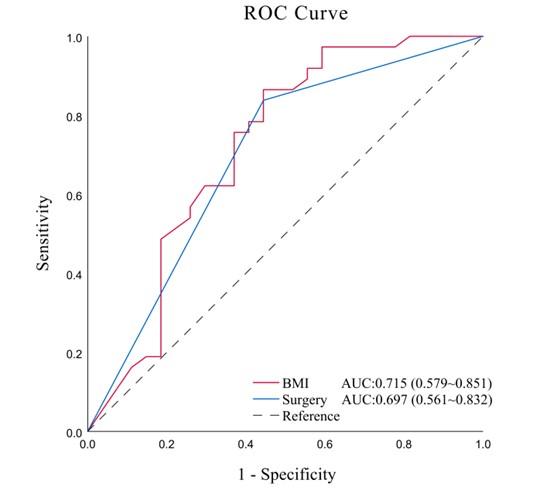

Supplement: Supplementary Figure 1 — ROC curve for the impact of baseline BMI and history of metabolic surgery. [file Image1.jpeg]
